# Comparison of Acuros (AXB) and Anisotropic Analytical Algorithm (AAA) for dose calculation in treatment of oesophageal cancer: effects on modelling tumour control probability

**DOI:** 10.1186/s13014-014-0286-3

**Published:** 2014-12-23

**Authors:** Sriram Padmanaban, Samantha Warren, Anthony Walsh, Mike Partridge, Maria A Hawkins

**Affiliations:** Oxford Cancer Centre, Oxford University Hospitals, Oxford, OX3 7LE UK; CRUK/MRC Oxford Institute for Radiation Oncology, Gray Laboratories, University of Oxford, Oxford, OX3 7DQ UK

## Abstract

**Aim:**

To investigate systematic changes in dose arising when treatment plans optimised using the Anisotropic Analytical Algorithm (AAA) are recalculated using Acuros XB (AXB) in patients treated with definitive chemoradiotherapy (dCRT) for locally advanced oesophageal cancers.

**Background:**

We have compared treatment plans created using AAA with those recalculated using AXB. Although the Anisotropic Analytical Algorithm (AAA) is currently more widely used in clinical routine, Acuros XB (AXB) has been shown to more accurately calculate the dose distribution, particularly in heterogeneous regions. Studies to predict clinical outcome should be based on modelling the dose delivered to the patient as accurately as possible.

**Methods:**

CT datasets from ten patients were selected for this retrospective study. VMAT (Volumetric modulated arc therapy) plans with 2 arcs, collimator rotation ± 5-10° and dose prescription 50 Gy / 25 fractions were created using Varian Eclipse (v10.0). The initial dose calculation was performed with AAA, and AXB plans were created by re-calculating the dose distribution using the same number of monitor units (MU) and multileaf collimator (MLC) files as the original plan. The difference in calculated dose to organs at risk (OAR) was compared using dose-volume histogram (DVH) statistics and p values were calculated using the Wilcoxon signed rank test. The potential clinical effect of dosimetric differences in the gross tumour volume (GTV) was evaluated using three different TCP models from the literature.

**Results:**

PTV Median dose was apparently 0.9 Gy lower (range: 0.5 Gy - 1.3 Gy; p < 0.05) for VMAT AAA plans re-calculated with AXB and GTV mean dose was reduced by on average 1.0 Gy (0.3 Gy −1.5 Gy; p < 0.05). An apparent difference in TCP of between 1.2% and 3.1% was found depending on the choice of TCP model. OAR mean dose was lower in the AXB recalculated plan than the AAA plan (on average, dose reduction: lung 1.7%, heart 2.4%). Similar trends were seen for CRT plans.

**Conclusions:**

Differences in dose distribution are observed with VMAT and CRT plans recalculated with AXB particularly within soft tissue at the tumour/lung interface, where AXB has been shown to more accurately represent the true dose distribution. AAA apparently overestimates dose, particularly the PTV median dose and GTV mean dose, which could result in a difference in TCP model parameters that reaches clinical significance.

## Background

Definitive chemoradiation (dCRT) plays an important role in the treatment of oesophageal cancer: for both squamous cell and non-operable adenocarcinoma patients, it offers a clear benefit compared to single modality treatment [1]. Recent evidence also suggests a survival benefit when CRT is used pre-operatively [2]. However, risks of local recurrence are high, with a recent study demonstrating that around 75% of recurrences in dCRT occur in the primary tumour [3], and improvement of loco-regional control appears to be a key factor in successful treatment for these patients. The introduction of advanced radiotherapy techniques, such as intensity modulation, has generated a renewed interest in dose escalation for oesophageal cancer, as delivery of a higher dose to the primary tumour, whilst maintaining dose to surrounding organs at risk at safe levels, may now be possible [4,5]. A systematic review of pre-operative CRT by Geh suggested that a radiation dose response exists for oesophageal cancer, and therefore that pathological tumour response would be improved if the radiotherapy dose were increased above ~ 50 Gy [5,6]. An investigation of the dose response would be supported by accurate knowledge of the dose delivered to the tumour. The dose delivered to normal tissues, such as lung and heart, will also be important in determining the extent of dose escalation possible for these patients.

A critical component in the analysis of the dose–response of oesophageal tumours is therefore the accuracy of the calculation of the dose distribution, which can be particularly challenging in the case of thoracic tumours, due to the presence of low density lung tissue surrounding the target volume. Advanced (‘type b’) dose calculation algorithms (such as AAA - Anisotropic Analytical Algorithm from Varian) now routinely available in commercial treatment planning systems show improved accuracy compared to the previous pencil beam (‘type a’) algorithms, but significant errors still persist at the lung/soft tissue interface, particularly in the re-build-up region [7,8]. The Acuros (AXB) algorithm, recently introduced in the Eclipse treatment planning system (Varian Medical Systems, Palo Alto, USA) [9] iteratively solves the Linear Boltzman Transport Equation and has been demonstrated to show equivalent accuracy to Monte Carlo calculations in heterogeneous media [10,11]. Previous studies comparing the use of AXB vs AAA for calculating dose in patients, e.g. for breast [12], lung [13-15], and nasopharynx [16] have shown that significant differences in the calculated dose deposition may arise when the planning target volume (PTV) encompasses a range of tissue types, or is close to the interface between different density materials. Observed differences in mean PTV dose are up to 1.2% in lung [13], 1.6% in muscle [12] and 2% in bone [16], where differences in minimum PTV dose of up to 4% have been observed [16].

This study therefore seeks to compare the AXB and AAA dose calculation algorithms specifically for radiotherapy planning of thoracic oesophageal tumours, where the radiation dose in the lung/soft tissue interface region may be important in modelling both tumour control and normal tissue complications.

## Methods

### Treatment planning

10 patients with mid-oesophageal cancer treated with definitive chemoradiotherapy were identified retrospectively. In conformance with our institutional consent procedure, all patients prospectively gave written consent for their imaging data to be used for education and research purposes. This work was approved as part of on-going radiotherapy physics projects by the appropriate local review board. CT images with IV contrast were acquired with a slice thickness of 2.5 mm during ‘quiet’ breathing and imported into Eclipse v10 for contouring and treatment planning. Gross tumour volume (GTV) including primary tumour and nodal disease was delineated with the aid of PET/CT and endoscopic ultrasound where available. Margins of 10 mm axially and 20 mm superior/inferior were added to create the clinical target volume (CTV), and the PTV was generated by adding a 10 mm margin in all directions according to institutional protocol. For the studied patient subset, the mean PTV was 337 cm^3^ (range 140–658 cm^3^). The PTV was further divided into soft tissue ‘PTV_tissue’ and ‘PTV_lung’ where the PTV intersected with lung tissue. Normal tissues (heart, lungs, liver and cord) were delineated, with an additional planning organ at risk (CordPRV) volume created using a 5 mm margin around the cord.

Conformal (3D) and RapidArc (RA) treatment plans were calculated for each patient with a 2.5 mm isotropic dose grid, a dose prescription of 50 Gy/25 fractions and were initially optimised using AAA version 10.0.28 with the aim of covering 95% of PTV with the 95% isodose contour. 3D_AAA plans consisted of 3–4 beams (anterior, two lateral oblique and post), normalised to 50 Gy at the reference point. RA_AAA plans of 2 full arcs with collimator rotation ± 5-10° and were normalised so that the median PTV dose was 50 Gy. AXB plans (3D_AXB and RA_AXB) were then re-calculated as dose to medium with Acuros version 10, using the same MU and MLC as for the AAA plans. The re-calculated AXB plan is believed to be a more accurate representation of the dose delivered to the patient compared to the AAA optimised treatment plans currently used in clinical routine.

### Plan analysis

**Figure 1:**
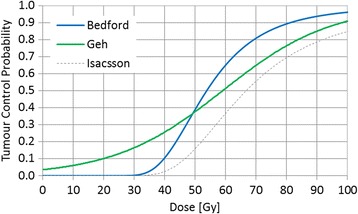
**Comparison of TCP models for oesophagus based on published parameters from different studies.**

Dose-volume parameters for target volumes (GTV mean dose, PTV D_95%_, PTV median and PTV max dose and mean dose for PTV_Lung and PTV_tissue) were compared for all 10 patients. Additionally, organ at risk dose metrics (CordPRV D_max_, Lung mean dose and V_20Gy_, heart mean dose, V_25Gy_ and V_40Gy_ and liver mean dose) were recorded. The values for 3D_AAA vs 3D_AXB and RA_AAA vs RA_AXB plans were compared using the Wilcoxon signed rank test, where average differences between the paired datasets are specified as median, (range: min - range: max) with p < 0.05 taken as significant. Tumour control probability (TCP) for the primary tumour was calculated for all plans via the differential DVH for the GTV exported in bins of 0.25 Gy, using 3 different models taken from the literature (Figure 1).

Briefly, the Geh TCP [6] model is logistic regression fit to pathological complete response data from 26 chemoradiotherapy trials for pre-operative oesophageal cancer:

$$ TCP(z)=\frac{ \exp (z)}{1+ \exp (z)} $$

and where the protocol prescribed doses of radiotherapy and chemotherapy (5FU and cisplatin) were used as variables:

$$ \begin{array}{l}z={a}_0+{a}_{1\ } total\ RT\  dose+{a}_2\  total\ RT\  dose\times dose\kern0.5em  per\kern0.5em  fraction\ \\ {}+{a}_3\kern0.5em  duration+{a}_4\kern0.5em  age+{a}_5\ 5FU\  dose\\ {}+{a}_6\kern0.5em  cisplatin\  dose\kern0.75em \end{array} $$

The Bedford TCP [17] calculation uses the Webb-Nahum model [18] which assumes a normal distribution of α the LQ parameter, with standard deviation σ_α_. Values used are σ_α_ = 0.08 Gy^−1^ and α = 0.40 Gy^−1^ chosen to fit observed local control rates for oesophageal cancer [19]. An alternative linear LQ TCP model (Isaacson [20]) using historical data (prior to the CT radiotherapy planning era) has also been used, although uncertainties in the dose distribution data for this model means the absolute TCP values may be less relevant to current chemoradiotherapy treatments, it is included for comparison of predicted relative differences in TCP.

## Results

### Dose to GTV/PTV

**Figure 2:**
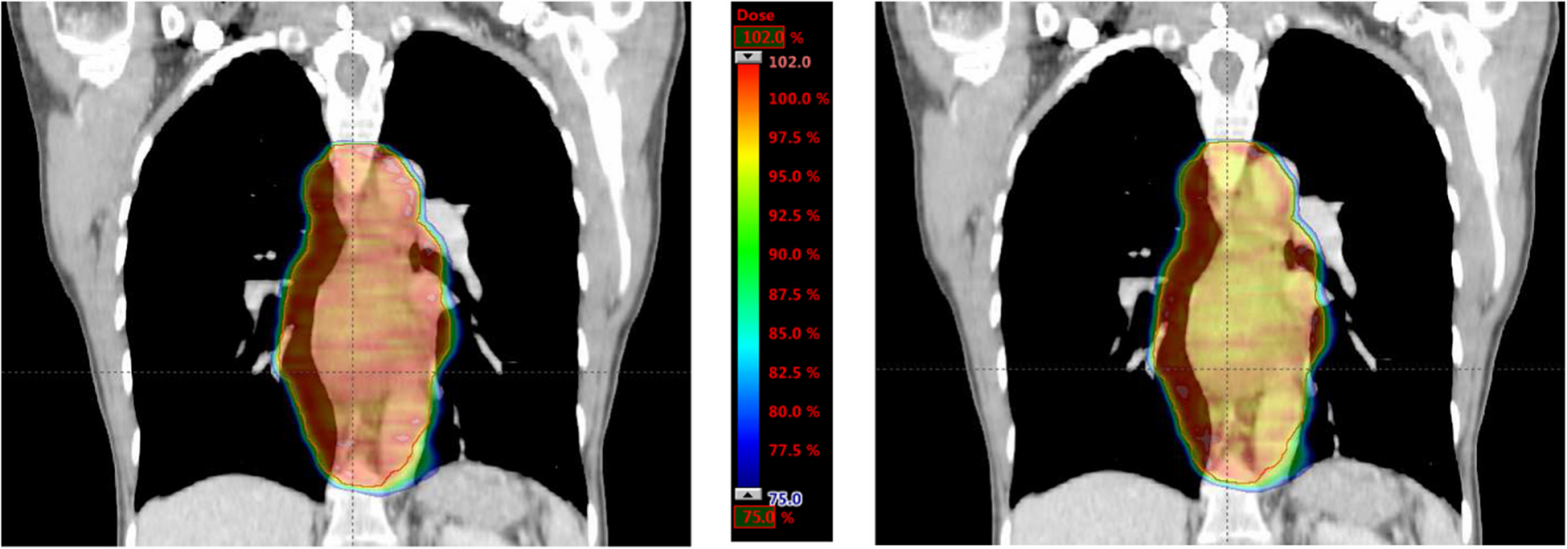
**Comparison of dose distribution calculated using AAA and AXB dose calculation algorithms for mid-oesophageal cancer.** Left: Coronal slice of RapidArc plan calculated using AAA showing the dose distribution for a mid-oesophageal cancer patient with the largest proportion of lung tissue in the PTV (red contour, total size of 529 cm3). The dose colour scale ranges from 75% (blue) to 102% (red) of the prescribed dose of 50 Gy. Right: The same RapidArc plan recalculated using the AXB dose calculation algorithm (for the same patient, with the same dose scale) showing a reduction in dose, particularly in soft tissue in the interior of the PTV.

**Figure 3:**
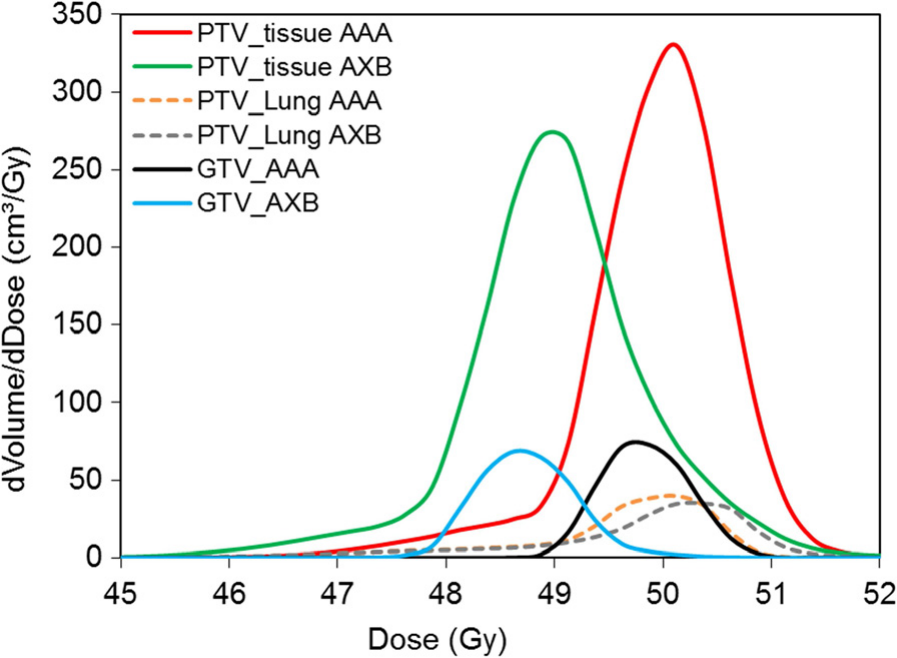
**Differential DVH for GTV and PTV using AAA and AXB dose calculation algorithms.**

A comparison of the dose distribution for RapidArc plans calculated using the AAA and AXB dose algorithms for a patient with a PTV of 529 cm^3^ is shown in Figure 2, indicating the lower dose obtained with the more accurate AXB dose calculation algorithm, particularly in the interior of the PTV. The differences in dose to GTV, and to the lung and tissue components of the PTV, are given in Table 1 (averaged across all ten patients). It appears that less dose is actually delivered to GTV when re-calculated using AXB, as compared to the dose predicted by AAA. For RA plans, the difference in mean dose to GTV is on average 1.0 Gy (0.3 - 1.5 Gy; p < 0.05) and the difference in PTV median dose is on average 0.9 Gy (0.5 – 1.3 Gy; p < 0.05). As shown in Table 1 and Figure 3, the difference in calculated dose to the PTV_Lung tissue using AAA or AXB for RapidArc plans is not statistically significant. However, a difference of (on average) 0.9 Gy [0.5 – 1.3 Gy] less dose in the soft tissue region of the PTV (PTV_tissue) is found when using the AXB algorithm as compared to AAA. This indicates that the difference in the two dose calculation algorithms is more than a simple MU normalisation factor, and can lead to differences in relative dose distribution. Similar results for 3D conformal plans are shown in Tables 1 and 2, indicating the same apparent overestimation of dose to the soft tissue components of the target when calculated with the AAA algorithm.

**Table 1:** **Comparison of target volume dose calculated using AAA or AXB for all patients for a) RapidArc plans and b) 3D conformal radiotherapy (CRT) plans**

**a)**			
**Target (dose metric)**	**Mean dose [min, max] in Gy**		**p**
	**RA_AAA**	**RA_AXB**	
	**CRT_AAA**	**CRT_AXB**	
GTV (mean)	49.9 [49.6, 50.0]	48.8 [48.3, 49.6]	0.01
PTV (D_95%_)	48.7 [48.0, 49.1]	47.7 [47.1, 48.2]	0.01
PTV (median)	50.0 (normalisation)	49.1 [48.7, 49.5]	0.01
PTV (max)	52.3 [51.5, 53.1]	53.1 [52.5, 53.9]	0.01
PTV_Lung (mean)	49.6 [49.2, 49.8]	49.7 [49.0, 50.1]	0.18
PTV_tissue (mean)	49.9 [49.8, 50.0]	49.0 [48.7, 49.5]	0.01
**b)**			
**Target (dose metric)**	**Mean dose [min, max] in Gy**		**p**
	**CRT_AAA**	**CRT_AXB**	
GTV (mean)	50.6 [49.8, 51.2]	49.6 [49.0, 50.1]	0.01
PTV (D95%)	48.2 [45.7, 49.4]	47.4 [44.9, 48.6]	0.01
PTV (median)	50.2 [49.6, 50.7]	49.2 [48.1, 49.7]	0.01
Norm point	50.0 (normalisation)	48.8 [48.0, 49.7]	
PTV (max)	52.9 [51.9, 53.8]	53.4 [52.4, 54.8]	0.01
PTV_Lung (mean)	49.4 [47.8, 50.3]	49.4 [47.7, 50.7]	0.12
PTV_tissue (mean)	50.2 [49.5, 50.7]	49.2 [48.4, 49.8]	0.01

**Table 2:** **Mean % dose difference (quoted as a percentage of the absolute value calculated with AAA) to organs at risk calculated using AAA or AXB for a) RapidArc plans and b) 3D conformal radiotherapy (CRT) plans**

**a)**		
**OAR and dose metric**	**Mean difference [min, max] % (RA_AAA) – (RA_AXB)**	**p**
Cord PRV D_max_	−3.0% [−3.9%, −1.6%]	<0.05
Lung Mean	−1.7% [−5.2%, −0.4%]	<0.05
Lung V_20Gy_	−2.7% [−8.3%, 0.0%]	<0.05
Heart Mean	−2.4% [−3.0%, −1.2%]	<0.05
Heart V_25Gy_	−5.7% [−7.4%, −4.5%]	<0.05
Heart V_40Gy_	−3.6% [−8.3%, −1.3%]	<0.05
Liver Mean	−1.2% [−2.2%, 0.0%]	NS
**b)**		
**OAR and dose metric**	**Mean difference [min, max] % (CRT_AAA) – (CRT_AXB)**	**p**
Cord PRV D_max_	−2.0% [ −8.2%, 2.7%]	<0.05
Lung Mean	−2.2% [ −4.5%, 0.3%]	<0.05
Lung V_20Gy_	−1.5% [ −3.7%, 0.4%]	<0.05
Heart Mean	−2.6% [ −3.4%, −1.7%]	<0.05
Heart V_25Gy_	−3.0% [−12.4%, −0.3%]	<0.05
Heart V_40Gy_	−6.9% [−18.4%, −2.2%]	<0.05
Liver Mean	−1.5% [ −2.5%, 0.6%]	NS

### Dose-volume histogram analysis/OAR

For every patient, OAR mean dose was also lower in the AXB recalculated plan than the AAA plan with the average dose difference (quoted as a percentage of the absolute value calculated with AAA) for RA plans as follows: lung 1.7%, heart 2.4% (Table 2) 3D CRT plans gave similar results (Table 2). The average difference in the volume of heart receiving a moderate dose (V_25_ Gy) was 5.7% and the higher dose volume in the heart (V_40_ Gy) was also significantly less by 3.6% on average for the RA_AXB dose calculation algorithm compared to the RA_AAA plan. 3D CRT plans showed a greater difference (of up to 18.4% for one patient) for heart V_40Gy_ with AXB. The CordPRV dose showed a significant reduction, on average of 3.0% for RA and 2.0% for 3D CRT, when re-calculated using the AXB dose algorithm to obtain a more accurate representation of the dose truly delivered to each patient.

### TCP analysis

An apparent difference in TCP expressed here as median [range: min, range: max] was found for all patients when re-calculating plans using AXB, due to the apparent overestimation of GTV dose by AAA. The magnitude of this difference depended on the choice of TCP model. Geh predicted a 1.2% [−0.4%, +1.9%] difference in local control from (RA_AAA) to (RA_ AXB) (p < 0.05), and the Bedford TCP model predicts a reduction of 3% [1.1%, 4.5%] from (RA_AAA) to (RA_ AXB) (p < 0.05). Similar values are seen for the 3D CRT plans: TCP Geh (CRT_AXB) is 1.4% [0.5%, 1.9%] different from the CRT_AAA mean TCP and Bedford CRT_AAA mean TCP changes by 3.1% [1.3%, 4.3%]. The Isacsson model TCP was 1.8% [0.7%, 2.7%] lower for both RA and CRT plans when comparing AXB to the AAA dose calculation algorithm.

## Discussion

This study compared the dose distributions obtained by two different dose calculation algorithms (AXB and AAA) for mid-oesophageal tumours. Lower doses were found when re-calculating AAA treatment plans with the AXB algorithm, and this dose difference was clinically significant for the GTV, PTV and certain organs at risk (heart, cord). The lower doses predicted by AXB compared to AAA are found for both 3D conformal and RapidArc plans, indicating that the differences in dose distribution for oesophageal cancer are not dependent on the beam delivery technique. This is in line with results from the literature, for example for lung tumours, where AXB predicts up to 1.1 Gy less than AAA to the soft tissue in the PTV for 3D, IMRT and RA plans [13]. Previous studies investigating the use of AXB in heterogeneous media [10,11,14] suggest that this algorithm is more accurate than the widely-used AAA, especially at the lung/soft tissue interface region [13,21,22]. In particular, AXB shows a much better agreement with Monte Carlo calculations [23] or dose measurements [24] in regions of re-buildup in soft tissue after the beam has passed through low density tissue such as lung or air. Our AXB results can therefore be considered as a better approximation of the true dose distribution than data obtained using AAA, and indicate an apparent over-estimation of dose to the primary tumour of around 1 Gy when calculated with AAA. The location of the dose over-estimation is particularly pertinent in the case of oesophageal cancers, where most local recurrences occur within the GTV, well inside the treatment field.

However, as most clinical outcome data is based on dose distribution calculated using “type b” (AAA) or “type a” (pencil beam) dose algorithms, the interpretation of these results in the context of current radiotherapy treatments and future dose escalation trials should be carefully analysed. Normalisation of 3D CRT radiotherapy treatment plans using AXB to meet the protocol dose prescription of 50 Gy would result in an increase in MU of around 2% (range 1.0% to 2.8%), with a corresponding increase in mean dose delivered to the surrounding normal tissues contained within the Body-PTV contour.

Alternative methods to account for the differences in dose distribution include creation of algorithm-specific radiobiological parameters, such as TCP models of local control in breast cancer with specific pencil beam, AAA or AXB parameters [25]; or re-analysis of the fitting parameters to take into account the improved dose calculation. This latter approach has been used in a study of dose-volume effects in radiation pneumonitis used for NTCP modelling of toxicity in lung [26]. The absolute TCP values from the Geh and Bedford models were broadly consistent at ~37%. The smaller TCP difference predicted by the Geh model (1.2% vs. 3% for Bedford) is likely to be due to the fact that the Geh model was derived from more heterogeneous population data.

It should be noted that optimisation of RA plans using AXB will, or course, produce plans which meet the prescribed dose constraints, but have a different MLC fluence. We have shown that this is not simply a scaled version of the AAA result (data not shown). One limitation of our current study is that we have compared dose distributions calculated using the planning CT dataset acquired during free-breathing. Studies using 4D-CT scans for dose accumulation throughout the respiratory cycle suggest that the dosimetric effect of movement is slight, even for small tumours entirely surrounded by low density lung [27]. The cranial-caudal location of the primary tumour in mid-oesophageal cancers can also be difficult to identify, although an analysis of fiducial marker movement during 4D-CT scans for mid-oesophageal cancer suggested 95% of intra-fractional tumour movement would be covered by a 7.4 mm margin in the cranial-caudal direction, and this is within our CTV to PTV margin of 10 mm. Variations in inter-fractional displacement have also been investigated using repeated 4D-CT scans [28], and indicate a general trend for tumour regression during the course of radiotherapy, but no systematic displacement was observed for mid-oesophageal tumours even after 20 fractions of radiotherapy treatment.

## Conclusions

Recalculating treatment plans for mid-oesophageal cancer using the more accurate AXB dose calculation algorithm instead of AAA produces a lower dose to the PTV and GTV, and lower mean dose to the surrounding organs at risk. The difference in dose is largest in areas of soft tissue surrounded by low density lung (such as the oesophageal primary tumour volume and the heart). Modelling dose–response from clinical trial data should specify the dose calculation algorithm used, in order to avoid bias in the generation of dose-volume constraints and radiobiological model parameters.
